# Larvicidal, adulticidal, and oviposition-deterrent activity of *Piper betle* L. essential oil to *Aedes aegypti*

**DOI:** 10.14202/vetworld.2019.367-371

**Published:** 2019-03-04

**Authors:** Riesna Martianasari, Penny Humaidah Hamid

**Affiliations:** Faculty of Veterinary Medicine, Universitas Gadjah Mada, Jl. Fauna No. 2 Karangmalang 55281, Yogyakarta, Indonesia

**Keywords:** adulticide, *Aedes aegypti*, larvicide, oviposition, *Piper betle* L

## Abstract

**Background and Aim:**

*Aedes aegypti* is a primary vector of many arthropod-borne diseases. One of the diseases, dengue fever, is an endemic disease in Indonesia causing high mortalities for decades. There are no preventive and specific treatments for dengue so far. Therefore, prevention of this disease largely depends on the mosquito control. Since resistance to chemical insecticides occurred worldwide, the study on alternate and new mosquito insecticides are mandatory. This study aimed to demonstrate the effect of essential oil from *P. betle* L. in the larval and adult stages, as well as its influence on oviposition activity of *A. aegypti* mosquito.

**Materials and Methods:**

*P. betle* efficacy was evaluated in various stages of *A. aegypti* development. For the larvicidal activity, larvae instar III stage was used. Adulticidal assay in this experiment was performed using newly emerged *A. aegypti*. For oviposition assay, mated *A. aegypti* was tested for their responses to *P. betle*-treated and non-treated ovitraps.

**Results:**

*P. betle* L. - adulticide activity was effective with a concentration of 2.5 μl/ml, caused 100% mortality within 15-30 min. Larvicide activity was observed after 1 h, 24 h, and 48 h post-treatment with LC_50_183, 92.7, and 59.8 ppm and LC_90_ 637, 525, and 434.7 ppm, respectively. Oviposition activity index was −0.917 in 1000 ppm. In addition, the eggs number of *A. aegypti* oviposition with 100 ppm of essential oil *P. betle* L. was 5 times lower than the control.

**Conclusion:**

This study demonstrated clearly that essential oil derived from *P. betle* L. potentially acts as alternate bioinsecticide to control *A. aegypti* population. The application can be varied or combined in different stages of mosquito development.

## Introduction

*Aedes aegypti* is the principal vector of many arthropod-borne diseases in tropical areas worldwide. *A. aegypti* efficiently transmits dengue fever (DF) due to its anthropophilic feeding and breeding characteristics [1]. It has been reported that 71,668 DF human cases have so far occurred in Indonesia in 2015 covering 34 provinces which means that DF has spread now into all national territories [2].

Insecticides have been applied for control of mosquitoes in decades. Major chemical insecticides distributed are pyrethroids, organophosphates (OPs), carbamates, and organochlorines [3]. In the context of mosquito insecticides, pyrethroids become widely used in many forms of derived compounds, for coils, repellents, bed nets, and also space spraying formulas. Since mosquito control strategies are mainly based on the use of insecticides, resistance development can be expected to occur in frequently exposed *A. aegypti* populations. Several reports showed insecticide resistance development in different regions in Indonesia such as Bali, Java, and Kalimantan [4-8] to mainly pyrethroids and OPs. Botanical insecticides are considered to be environmentally friendly and safe for other organisms [9]. No studies have shown resistance to botanical-based insecticides among vector due to the limited use of botanical remedies in the control programs [10]. Plant extracts or phytochemical is the potential sources of commercial mosquito bioactive compound. Therefore, studies of herb-based compound should be importantly performed to provide new, safe, and effective insecticides. In addition, experiment using local herbs may rise the value of local herbs easily grown in the area and give the guarantee of sustainable production in the future.

The plant being natural sources of various compounds are known to contain mosquitocidal agents, which may act independently or in combination. Some phytochemical substances act as general toxicant against the adult and larvicidal, while others act as repellent or attractant which is interfering the growth and development, with the production of olfactory stimuli [11]. *Piper betle* L. has been used traditionally in Indonesia as herbal medicine. This plant contains bioactive compound such as terpinen-4-ol, safrole, eugenol, hydroxylchavicol, and *eugenyl acetate* which may have a repellent effect against mosquitoes [12].

This study aimed to demonstrate the effect of essential oil from *P. betle* L. in the larval and adult stages, as well as its influence on oviposition activity of *A. aegypti* mosquito.

## Materials and Methods

### Ethical approval

All of the procedures of this experiment were approved by the committee of ethics for preclinical research of LPPT Gadjah Mada University, Yogyakarta No. 00076/04/LPPT/VI/2017.

### Mosquito collection and rearing

*A. aegypti* eggs were collected in the area of Sleman, Yogyakarta Province, Indonesia. The eggs were obtained from several locations such as Depok (S07°45.967’E110°22.916’), Seyegan (S07°43.423’E 110°18.745’), Godean (S07°76.9720’E110°29.3890’), and Mlati (S07°45.667’E110°22.303’) where reportedly having high cases of dengue hemorrhagic fever [13]. Ovitraps were made from glass with black stain outside. A filter paper was placed in the mouth of the glass. Filled water and filter papers were replaced every week during collection dates. Samples were collected from April to September 2017. The collection sites were randomly selected with emphasis on previous DF cases and were regular fogging activities occurred in those urban areas. Egg-containing papers were dried at room temperature and stored in plastic containers. The eggs were identified microscopically with its elongated oval shaped with bright black shell [14]. Eggs were hatched in the insectary of the Department of Parasitology, Faculty of Veterinary Medicine, Gadjah Mada University. Furthermore, chicken liver (wet and dried) was fed during larvae development until pupa stages. Adult mosquitoes reared from these collected eggs were then fed with 10% sugar solution absorbed into cotton balls.

### Essential oil of P. betle L

*P. betle* L. leaves were obtained locally from herbal medicine garden in Seyegan, Yogyakarta Province, Indonesia. Determination of *P. betle* L. was performed in the Laboratory of Plant Systematics, Faculty of Biology, Gadjah Mada University, with the identification number of 01244/S. Tb./II/2018.

Essential oil extraction was performed in the Laboratory of Industrial Engineering and Side Product Control, Faculty of Agricultural Technology, Gadjah Mada University, Yogyakarta. Extraction process to produce essential oil was performed by water-steam distillation method [15]. About 3 kg of *P. betle* L. leaves were dried for 8 h in a drying cabinet at 50°C. Dry leaves were steamed on boiled water (100°C) for 4-5 h. Essential oil was produced by passing the vapors into water-steam distillation in a Clevenger-type apparatus. Essential oil produced was stored in sterile a glass container. Total essential oil produced was 1.6 ml with golden yellow and typically *P. betle* L. odor.

### Larvicidal assay

For each larvicidal activity, five larvae instar III were used in triplicate. The concentrations of essential oil *P. betle* L. used were 1000 ppm, 500 ppm, 100 ppm, 50 ppm, 10 ppm, and 1 ppm. Essential oil was prepared as stock in aquadest. 25 ml of each concentration was put onto a100-ml cup container and 25 ml of aquadest without essential oil was served as control. The larvae mortality was observed within 1 h, 24 h, and 48 h. Percent mortalities were counted using the formula: Mortality (%) = (Number of dead larvae/number of larvae introduced) × 100% [16]. The data were then processed using regression analysis to obtain LC_50_ and LC_90_ values [17].

### Adulticidal assay

Adulticidal assay in this experiment adapted CDC protocol and was performed to determine time for an insecticide to penetrate a mosquito. Doses and coating process of the bottles applied in this study were based on CDC standard [18]. Briefly, 1 ml of stock solution was diluted with ethanol as solvent to get desired concentrations. Five different concentrations were used in the experiment, i.e., 2.5 µl/ml, 2 µl/ml, 1.5 µl/ml, 1 μl/ml, and 0.5 µl/ml according to the previous studies [19]. Each subsequent concentration was inserted in CDC bottle for the coating [20] and one control bottle containing 1 ml of ethanol served as control. After the coated bottle was ready for the test (dry), 10-20 unfed mosquitoes and aged from 2 to 5 days old were taken by an aspirator. Then, mosquitoes were gently blown into the bottle. All the CDC susceptible tests were performed in triplicates. Mosquitoes were characterized die if cannot stand, walk, fly, or the wings were dislodged [21]. The number of dead or alive mosquitoes was monitored at different time intervals, i.e., 15 and 120 min. The mosquito mortality data obtained were then processed using non-linear regression analysis to obtain LC_50_ and LC_90_ values [17].

### Oviposition assay

For oviposition assay, 50 *A. aegypti* that has been mated transferred in one test cage. 10% of sugar was placed inside test cage and blood by given by mice inserted overnight to induce ovulation of mosquitoes. Furthermore, two kinds of ovitraps were placed into the cage, ovitrap containing 2 ml 1% essential oil from stock solution and control ovitrap with only aquadest. Total volume of all ovitraps was 200 ml. Female mosquitoes were allowed to be in the testing cage for 1-2 days. Filter papers from each ovitrap were taken and the number of mosquito eggs produced was calculated under dissection microscope [10]. The oviposition test was repeated 2 times.

### Statistical analysis

The data presentation and analysis were processed using GraphPad Prism 7.02 software (GraphPad Software Inc., USA).

## Results

The effectiveness of *P. betle* L. essential oil on the larval stage of *A. aegypti* can be shown in Table-1. Mortalities were observed in the concentration of 5 ppm-1000 ppm which was increased following increasing concentration. Larval mortality within 24 h at concentrations of 5 ppm-1000 ppm was 6.7%, 12.3%, 13.3%, 35.3%, and 100%, respectively (Table-1). Aquadest control showed no mortality until 48 h observation. Percent mortality of *A. aegypti* to *P. betle* L. was a dose-dependent response. LC_50_ value of the essential oil of *P. betle* L. at observation of 1 h, 24 h, and 48 h was 183, 92.7, and 59.8 ppm, respectively, while the LC_90_ was 637, 525, and 434.7 ppm after 1 h, 24 h, and 48 h (Table-2).

**Table-1:** Mortality of *A. aegypti* larvae using essential oil *Piper betle* L.

Time	Percent mortality of larvae±SD							Control

	Concentration (ppm)							

	1	5	10	50	100	500	1000	
1 h	0±0.0	0±0.0	0±0.0	0±0.0	6.7±11.5	100±0.0	100±0.0	0*
24 h	0±0.0	6.7±11.5	12.3±10.8	13.3±11.5	35.3±45	100±0.0	100±0.0	0*
48 h	0±0.0	13.3±11.5	19±1.7	20±0.0	55.3±48	100±0.0	100±0.0	0*

CI=Confidence interval 95%, SD=Standard deviation,

* =No mortality, *A. aegypti*=*Aedes aegypti*

**Table-2:** LC_50_ and LC_90_ values of *Aedes aegypti* mosquito larvae using essential oil *Piper betle* L.

Time	LC_50_ (ppm)	LC_90_ (ppm)	Slope±SE
1 h	183	637	34.94±12.26
24 h	92.7	525	36.2±7.752
48 h	59.8	434.7	36.1±5.992

CI=Confidence interval 95%, SE: Standard error

**Table-3:** Toxicity of *Piper betle* L. essential oil to adult *A. aegypti*.

Concentration (μl/ml)	Percent mortality (M±SD)				LC_50_ 95%	LC_90_ 95%

	30 min	60 min	90 min	120 min		
2.5	100	100	100	100	0.955	1.485
2	10±13.2	55±20.2	85±8.6	100		
1.5	10±12.5	50±28.4	80±20.8	90±10		
1	0	7±11.5	30±14.4	50±5.7		
0.5	5±0.0	5±0.0	5±0.0	5±0.0		
Control	0	0	0	0		

M=Mean in triplicates, SD=Standard deviation, *A. aegypti*=*Aedes aegypti*

The toxicity of *P. betle* L. essential oil to adult *A. aegypti* is shown in Table-3. The most effective concentration of essential oil *P. betle* L. which caused 100% mortality with the fastest time (30 min of exposure) was 2.5 µl/ml concentration. At 30 min exposure, concentrations of 0.5 µl/ml-2 µl/ml show mortality <50%. After 60 min exposure, 2 µl/ml and 1.5 µl/ml concentrations showed 55% and 50% mortality rates, respectively. No difference in mortality was observed (5±0, M±standard deviation) in 0.5 µl/ml concentration with various exposure time until 2 h. No mortality in the control group was observed during the experiment. The values of LC_50_ and LC_90_ obtained for 2 h adulticide assay were 0.955 and 1.485 µl/ml (confidence interval 95%).

Oviposition assay with essential oil of *P. betle* L. showed a significant effect compared to control. The mean of total eggs produced on three replications at each treatment and control was 125.3 and 623.6 eggs, respectively. Essential oil *P. betle* L. at a concentration of 100 ppm showed a significant difference (p≤0.05; t=10.37; *df*=2) on the number of eggs produced compared to control using only tap water.

## Discussion

Larvicidal activity of essential oil from *Piperaceae* had been widely studied to control mosquito larvae. Essential oil of *Piper arboreum*, *Piper aduncum*, *Piper marginatum*, *Piper longum*, *Piper gaudichaudianum*, *Piper permucronatum*, and *Piper humaytanum* potentially used to control *Culex quinquefasciatus*, *Anopheles gambiae*, and *A. aegypti* [22,23]. Essential oil *Piper aduncum* showed larvicidal activity against *A. aegypti* mosquito larvae with 100% mortality at 500 and 1000 ppm concentration [24]. Similar concentrations of *Piper marginatum* essential oil also caused 100% mortality of *A. aegypti* larvae within a few hours [22]. The values of LC_50_ and LC_90_ in this study were smaller than other larvicidal studies using a crude extract of several herbs. The LC_50_ values for 24 h and 48 h were 236.73 ppm and 98.45 ppm using *P. betle* extract, 124.28 ppm and 95.75 ppm using *Areca catechu* extract, and 313.58 ppm and 122.9 ppm using *Nicotiana tabacum* extract [25]. It is known that the smaller the LC_50_ value, the greater the toxic potential or acute toxicity. However, this LC_50_ value only illustrates the potential for the toxicity of a relative toxin but does not represent the safe dose limits [26]. The result of this study also related to the fact that *A. aegypti* larval mortality is influenced by essential oil concentration and duration of exposure time [25]. Presumably, the larvicidal phytochemicals in this study affected on the midgut epithelium, then further affecting gastric ceca and malformed tubules in mosquito larvae, as discussed elsewhere [27]. Based on our result, suggested potential use of *P. betle* L. essential oil is at the concentration of 500 ppm to control *A. aegypti* larval stage. This concentration is higher than chemical insecticide commonly used (temephos) which can cause death at a dose of 0.1 mg/l in water [28]. However, resistance to temephos is widely reported in countries such as in Brazil [29], Argentina [30], Bolivia [31], Venezuela [32], Columbia [33], as well as in Indonesia [34]. On the other side, resistance of *A. aegypti* to *P. betle* L. is never reported so far. Presumably, the mosquito population is never exposed directly to the plant derivative compound in the field.

Adulticide effect in this study, which is represented by LC_50_ and LC_90_, is lower than of *Piper nigrum* previously studied [19]. The concentration of herbal extracts causes total mortality in adult mosquitoes to vary among plant species. Total mortality achieved by *Thymus vulgaris* (0.075 µl/ml), *Lippia adoensis* (0.75 µl/ml), *Moulla vaspicata* (0.75 µl/ml), *Chenopodium ambrosioides* (0.75 µl/ml), *P. nigrum* (1 µl/ml*), Eucalyptus citriodora* (10 µl/ml), *Nigella sativa* (2.5 µl/ml), *Ocimum lamiifolium* (5 µl/ml), *Eucalyptus globules* (7.5 µl/ml) and *Schinu*s *moll*e (7.5 µl/ml) [19]. *P. betle* L. adulticidal activity is comparable to *N. sativa* (2.5 µl/ml) to kill 100% adult *A. aegypti* mosquitoes. Died mosquitoes are definitely observed as paralysis to fly and only at the bottom of the bottle. The paralysis effect is known as knockdown (reversible and falling paralysis effect) post exposed with adulticidal compound [19]. Resistance to pyrethroid-based compounds is reported in several regions in Indonesia [4-7]. These pyrethroids are the main chemical insecticide widely used as adulticidal. The use of natural compounds such as *P. betle* L. in this experiment can be potentially used as an alternative bioinsecticide of adult *A. aegypti*.

The eggs number from oviposition assay with 100 ppm of *P. betle* L. was 5 times lower than the control (p≤0.05). Similarly, oviposition study using essential oil of *Etlingera elatior* showed decreased number of *A. aegypti* eggs at a concentration of 100 ppm significantly (p≤0.05) [35]. Some other essential oils such as *Cinnamon zeylanicum, Zingiber officinale*, and *Rosmarinus officinalis* are also reported having oviposition-deterrent activities to *Anopheles stephensi, A. Aegypti*, and *C. quinquefasciatus* [36]. Selection of oviposition sites by female mosquitoes is a major factor in determining the number of egg proliferation, population density, and geographical area differences [37]. *A. aegypti* have a habit of laying eggs following their visual and olfactory sensitivity to find an appropriate oviposition spot to distinguish the physical and chemical components of the water inside [38]. The effects of essential oil in water on female mosquitoes are presumably similar to the repellent effect on topical application to avoid mosquito bites, to potentially cause the mosquito not to lay eggs in the ovitrap provided.

## Conclusion

In this study, essential oil of *P. betle* L. could serve as an alternative option to control *A. aegypti* mosquitoes. This ubiquitous local flora of Indonesia has larvicidal, adulticidal, and oviposition-deterrent activities against *A. aegypti*.

## Authors’ Contributions

RM performed the experiments, analyzed the data, and wrote the manuscript. PHH designed the study, coordinated the work, and wrote the manuscript. Both authors read and approved the final manuscript.

## References

[1] WHO (2009). Dengue:Guidelines for Diagnosis, Treatment, Prevention and Control.

[2] Indonesia, MOHRO (2015). Demam Berdarah Biasanya Mulai Meningkat di Januari;2018.

[3] WHO (2007). Global Insecticide Use for Vector-Borne Disease Control.

[4] Hamid P.H, Prastowo J, Widyasari A, Taubert A, Hermosilla C (2017). Knockdown resistance (kdr) of the voltage-gated sodium channel gene of *Aedes aegypti* population in Denpasar, Bali, Indonesia. Parasit. Vectors.

[5] Hamid P.H, Prastowo J, Ghiffari A, Taubert A, Hermosilla C (2017). *Aedes aegypti* resistance development to commonly used insecticides in Jakarta, Indonesia. PLoS One.

[6] Sayono S, Hidayati A.P, Fahri S, Sumanto D, Dharmana E, Hadisaputro S, Asih P.B, Syafruddin D (2016). Distribution of voltage-gated sodium channel (Nav) alleles among the *Aedes aegypti* populations in central Java Province and its association with resistance to pyrethroid insecticides. PLoS One.

[7] Wuliandari J.R, Lee S.F, White V.L, Tantowijoyo W, Hoffmann A.A, Endersby-Harshman N.M (2015). Association between three mutations, F1565C, V1023G and S996P, in the voltage-sensitive sodium channel gene and knockdown resistance in *Aedes aegypti* from Yogyakarta, Indonesia. Insects.

[8] Hamid P.H, Ninditya V.I, Prastowo J, Haryanto A, Taubert A, Hermosilla C (2018). Current status of *Aedes aegypti* insecticide resistance development from Banjarmasin, Kalimantan, Indonesia. Biomed. Res. Int.

[9] Jbilou R, Amri H, Bouayad N, Ghailani N, Ennabili A, Sayah F (2008). Insecticidal effects of extracts of seven plant species on larval development, alpha-amylase activity and offspring production of *Tribolium castaneum*(Herbst) (Insecta:Coleoptera *Tenebrionidae*). Bioresour. Technol.

[10] Cheah S.X, Tay J.W, Chan L.K, Jaal Z (2013). Larvicidal, oviposition, and ovicidal effects of *Artemisia annua*(Asterales:Asteraceae) against *Aedes aegypti, Anopheles sinensis* and *Culex quinquefasciatus*(Diptera:Culicidae). Parasitol. Res.

[11] Elumalai D, Hemavathi M, Hemalatha P, Deepaa C.V, Kaleena P.K (2016). Larvicidal activity of catechin isolated from *Leucas aspera* against *Aedes aegypti, Anopheles stephensi* and *Culex quinquefasciatus*(Diptera:Culicidae). Parasitol. Res.

[12] Hodijah D.N, Widawati M (2014). Potential topical natural repellent against *Ae. aegypti, Culex* Sp *Anopheles* Sp. Mosquitoes. J. Health Sci. Indones.

[13] Jati P.S (2013). Model Backpropagation Neural Network Untuk Peramalan Kasus Demam Berdarah di D.I Yogyakarta. (Bachelor).

[14] Wall R, Shearer D (2001). Veterinary Ectoparasites:Biology, Pathology and Control.

[15] Baser K.H.C, Buchbauer G (2010). Handbook of Essential Oils:Science, Technology and Applications.

[16] Panneerselvan C, Murugan K, Kovendan K, Kumar P.M (2012). Mosquito larvacidal, pupicidal, adulticidal, and repellent activity of *Artemisia nilagirica*(Family:Compositae) against *Anopheles stephensi* and *Aedes aegypti*. Parasitol. Res.

[17] Finey D.J (1971). Probit Analysis.

[18] Brogdon W.G, Chan A (2010). Guideline for Evaluating Insecticide Resistance in Vectors Using the CDC Bottle Bioassay, Atlanta, USA.

[19] Massebo F, Tadesse M, Balkew M, Michael T.G (2013). Bioactivity of essential oil of local plants against adult *Anopheles arabiensis*(Diptera:Culicidae) in Ethiopia. Adv. Biosci. Biotechnol. J.

[20] CDC (2016). Guideline for Evaluating Insecticide Resistance in Vectors Using the CDC Bottle Bioassay.

[21] Perea E.Z, Leon R.B, Salcedo M.P, Brogdon W.G, Davine G.J (2009). Adaptation and evaluation of the bottle assay for monitoring insecticide resistance in disease vector mosquitoes in the Peruvian Amazon. Malar. J.

[22] Santana H.T, Trindade F.T.T, Stabeli R.G (2015). Essential oil of leaves of piper species display larvacidal activity against the dengue vector *Aedes aegypti*(Diptera *Culicuidae*). Rev. Bras. Plantas Med.

[23] Morais S.M, Facundo V.A, Bertini L.M, Cavalcanti E.S.B, Junior J.F.A, Ferreira S.A, Brito E.S, Neto M.A (2007). Chemical composition and larvicidal activity of essential oils from *Piper* species. Biochem. Syst. Ecol.

[24] Oliveira G.L, Cardoso S.K, Larajunior C.R, Vieira T.M, Guimares E.F, Figueiredo L.S, Martins E.R, Moreira D.L, Kaplan M.A (2013). Chemical study and larvacidal activity against *Aedes aegypti* of essential oil of *Piper aduncum* L. (Piperaceae). An. Acad. Bras. Cienc.

[25] Tennyson S, Arivoli S, Raveen R, Bobby M, Dhinamala K (2012). Larvicidal activity of *Areca nicotiana tabacum* and *Piper betle* Leaf extracts against the dengue vector *Aedes aegypti*(Culicidae). Int. J. Res. Biol. Sci.

[26] Donatus L.A (2005). Toksikologi Dasar.

[27] Usta J, Kreydiyyeh S, Bakajian K, Nakkash-Chmaisse H (2002). *In vitro* effect of eugenol and cinnamaldehyde on membrane potential and respiratory complexes in isolated rat liver mitochondria. Food Chem. Toxicol.

[28] Srinivasan P.V, Nathan S.S, Ponsarkar A, Thanigaivel A, Edwin E.S, Rani S.S, Chellappandian M, Pradeepa V, Escaline J.L, Kalaivani K, Hunter W.B, Duraipandiyan V, Al-Dhabi N.A (2017). Comparative analysis of mosquito (Diptera:Culicidae *Aedes aegypti* Liston) responses to the insecticide temephos and plant-derived essential oil derived from *Piper betle* L. Ecotoxicol. Environ. Saf.

[29] Lima J.B, Da-Cunha M.P, Silva R.C.D, Galardo A.K, Soares S.S (2003). Resistance of *Aedes aegypti* to organophosphates in several municipalities in the state of Rio de Janeiro and Espirito Santo, Brazil. Am. J. Trop. Med. Hyg.

[30] Llinas G.A, Seccacini E, Gardenal C.N, Licastro S (2010). Current resistance status to temephos in *Aedes aegypti* from different regions of Argentina. Mem. Inst. Oswaldo Cruz.

[31] Biber P.A, Duenas J.R, Almeida F.L, Gardenal C.N, Almiron W.R (2006). Laboratory evaluation of susceptibility of natural subpopulations of *Aedes aegypti* larvae to temephos. J. Am. Mosq. Control Assoc.

[32] Rodriguez M.M, Bisset J, Fernandez D.M, Lauzan L, Soca A (2001). Detection of insecticide resistance in *Aedes aegypti*(Diptera:Culicidae) from Cuba and Venezuela. J. Med. Entomol.

[33] Grisales N, Poupardin R, Gomez S, Gonzalez I.F, Ranson H, Lenhart A (2014). Temephos resistance in *Aedes aegypti* in Colombia compromises dengue vector control. PLoS Negl. Trop. Dis.

[34] Mulyatno K.C, Yamanaka A, Ngadino, Konishi E (2012). Resistance of *Aedes aegypti*(L.) Larvae to temephos in Surabaya, Indonesia. Southeast Asian J. Trop. Med. Public Health.

[35] Silva P.C.B, Dutra K.A, Santos G.K, Silva R.C.S, Lulek J, Pinheiro P.M, Navarro D.M.A (2016). Evaluation of the activity of the essential oil from an ornamental flower against *Aedes aegypti*:Electrophysiology, molecular dynamics and behavioral assays. PLoS One.

[36] Prajapati V, Tripathi A.K, Aggarwal K.K, Khanuja S.P.S (2005). Insecticidal, repellent and oviposition-deterrent activity of selected essential oils against *Anopheles stephensi, Aedes aegypti* and *Culex quinquefasciatus*. Bioresour. Technol.

[37] Tawatsin A, Asavadachanukorn P, Thavara U, Wongsinkongman P, Bansidhi J, Boonruad T (2006). Repellency of essential oils extracted from plants in Thailand against four mosquito vectors (Diptera:Culicidae) and oviposition deterrent effects against *Aedes aegypti*(Diptera:Culicidae). Southeast Asian J. Trop. Med. Public Health.

[38] Kumar S, Wahab N, Warikoo R (2011). Bioefficacy of *Mentha piperita* essential oil against dengue fever mosquito *Aedes aegypti* L. Asian Pac. J. Trop. Biomed.

